# Normative Knee Range of Motion for Children

**DOI:** 10.3390/life15071000

**Published:** 2025-06-24

**Authors:** Muhammad Uba Abdulazeez, Maryam Alhefeiti, Shahad Alhammadi, Hajar Alnuaimi, Aminu Sabo Abdullahi, Lobna Shaikhoun, Kamiar Aminian, Georgios Antoniou Stylianides, Kassim Abdulrahman Abdullah

**Affiliations:** 1 Department of Mechanical and Aerospace Engineering, United Arab Emirates University, Al Ain 15551, United Arab Emirates; 201990203@uaeu.ac.ae (M.U.A.); 202005514@uaeu.ac.ae (M.A.); 201702582@uaeu.ac.ae (H.A.); 201850083@uaeu.ac.ae (L.S.); 2 Emirates Center for Mobility Research, United Arab Emirates University, Al Ain 15551, United Arab Emirates; 3 Department of Automotive Engineering, Abubakar Tafawa Balewa University, Bauchi 0248, Nigeria; 4 Laboratory of Movement Analysis and Measurement, École Polytechnique Fédérale de Lausanne, 1015 Lausanne, Switzerland; kamiar.aminian@epfl.ch; 5 Department of Biology, United Arab Emirates University, Al Ain 15551, United Arab Emirates; 202012386@uaeu.ac.ae; 6 Institute of Public Health, United Arab Emirates University, Al Ain 15551, United Arab Emirates; aminutade@uaeu.ac.ae; 7 Exercise Science and Kinesiology, Juniata College, Huntingdon, PA 16652, USA; stylianides@juniata.edu

**Keywords:** range of motion, children, anthropometry, knee, injury

## Abstract

Children may suffer knee injuries due to motor vehicle crashes, sports, and falls. Additionally, children can suffer from rheumatic, neurological, musculoskeletal, and neuromuscular disorders which restrict joint movement. These types of injuries and disorders often result in knee joint impairment, thereby affecting joint mobility. Understanding the range of motion (ROM) of the pediatric knee is vital in diagnosing, examining, and treating these injuries and disorders. This study was undertaken to establish normative values for passive (PROM) and active (AROM) range of motion of the pediatric knee and to examine the effects of anthropometric and demographic factors on knee joint ROM. Normative reference values for both passive and active knee ROM were established for 295 children in the United Arab Emirates (Arab and South Asian ethnicity). The subjects’ PROM averaged 131.2° (117.2°, 140.2°) for boys and 132.8° (120.9°, 140.3°) for girls. Similarly, the observed PROM for children was 132.2° (118.6°, 141.2°), versus 130.8° (119.9°, 139.3°) for adolescents. Conversely, the subjects’ AROM averaged 129.3° (118.8°, 137.9°) for boys and 130.5° (120.9°, 137.4°) for girls. The observed AROM averaged 130.2° (119.5°, 137.8°) for children and 128.6° (121.5°, 137.4°) for adolescents. Significant differences in knee ROM based on ethnicity were identified. Additionally, significant correlations were observed between anthropometric factors and knee joint ROM. The gender and age-based normative values established in this study can be used in medical and vehicle safety analyses of knee injuries sustained by children as well as in the evaluation of knee joint impairments due to rheumatic, neurological, musculoskeletal, and neuromuscular disorders, thereby improving the outcomes of knee injuries and the treatment of joint impairments in children.

## 1. Introduction

Range of motion (ROM) measurement is a common method of assessing joint mobility. This helps in the diagnosis of joint impairments, the choice of the appropriate treatment, and the evaluation of the effectiveness of the treatment [1,2,3,4,5,6,7]. While studies have been conducted on knee ROM in children [1,2,4,7], the literature on knee ROM is mostly focused on children with disabilities [8,9,10,11,12,13,14,15].

Normative ROM reference values are essential for the evaluation and treatment of neurological and neuromuscular joint disorders [1,2,4,5,7]. Additionally, pediatric normative ROM reference values are important for the assessment of joint impairments in pathological and non-pathological children [1,2]. However, only a few studies were found to have established normative reference values for knee joint ROM in children [1,2,4,7,16,17]. Of these studies, only two were solely focused on children [1,2], with two other studies focused on neonates [16,17]. Additionally, the studies were limited to small to moderate sample sizes, either passive [1,7,16] or active [4] measurements only, or passive knee extension ROM only [2]. Furthermore, all these studies were conducted on children from western populations.

Importantly, ROM has been shown to be affected by race/ethnicity, cultural habits, and daily life activities [3,5,18]. Knee ROM has been observed to change over time [6,7,19,20,21,22]. Additionally, factors such as age, gender, occupational activities, and BMI have been established as significantly affecting knee ROM [7,19,21,23,24,25,26,27,28,29,30,31,32,33,34]. Furthermore, knee ROM differs significantly between children, adolescents, and adults [7,19,21,22,35].

Therefore, this study was undertaken to establish normative knee ROM reference values for children in the United Arab Emirates (Arab and South Asian ethnicity) by measuring both the active (AROM) and passive (PROM) range of motion of the pediatric knee in an adequate sample of children aged 3–12 years (divided into children and adolescent groups). This will provide a better understanding of the ROM of the pediatric knee, thereby enhancing the assessment of knee injuries sustained by children and their associated risk factors.

## 2. Materials and Methods

### 2.1. Sample Size Estimation

The sample size for this study was determined based on the ISO 15535 standard [36]. The minimum number of subjects sampled randomly n is given by:

(1) $$n={\left(\frac{1.96\times CV}{a}\right)}^{2}\times 1.534^{2}$$

where 1.96 is the critical value from a standard normal distribution for a 95% confidence interval, CV is the coefficient of variation, and a denotes the percentage of relative accuracy required. The values of CV given by Pheasant & Haslegrave [37] were used for this study.

(2) $$n={\left(\frac{1.96\times 11}{2}\right)}^{2}\times 1.534^{2}=273.5=274\;subjects$$

Based on Equation (2), a minimum sample size of 274 children was required for this study to attain 95% confidence and 2% relative accuracy.

### 2.2. Study Participants

Two hundred and ninety-five children (295) from Al Ain city in Abu Dhabi, United Arab Emirates (UAE) participated in the study. The children were recruited between August 2022 and September 2023 from one public and two private schools, as well as from the UAE University Falaj housing. The inclusion criteria comprised: (i) children aged between three to twelve years; (ii) not having any rheumatic, neuromuscular, musculoskeletal, or neurological impairment. Conversely, the exclusion criteria were having any disorder that can inhibit normal joint motion. Ethical approval was obtained from the UAE University’s Social Science Research Ethics Committee (ERSC_2022_704). Written informed consent was obtained from the children’s parents/guardians, and assent was obtained from the children before the measuring process started. Age, gender, and ethnicity were recorded for each participant. Ethnicity was classified into 3 categories: Arab, South Asian, or “Other”, based on the ethnic composition of the UAE. The age categories include 3–9 years (children) and 10–12 years (adolescents) according to the World Health Organization classification [38]. The children were divided into two age groups in order to analyze the effects of age on normative knee ROM in children.

### 2.3. Range of Motion Measurement

The active and passive knee joint ROM was measured in the children by means of an electro-goniometer (Vernier, Beaverton, OR, USA) based on the protocol established by Norkin and White [19]. The validity of goniometry in assessing extremity joint ROM has been reported in several studies [39,40,41]. Additionally, its validity in measuring knee joint ROM has also been reported in several studies [42,43,44,45], which have shown high validity. Therefore, it was not within the scope of our study to evaluate its validity.

The study was performed at schools during normal school hours. Although all the measurements were taken during the same time period for all the children (between 9 am and 1 pm), the time-of-day reproducibility of the measurements was not evaluated, as it was beyond the scope of our study. Finally, information regarding the level and type of physical activity affecting joint mobility was not captured during the course of this study. A trained research assistant performed the passive ROM measurements and assessed the active measurements. All the measurements were obtained from the left knee of the children and conducted in the supine position to ensure consistency throughout the study. Additionally, the publicly available dataset of extremity joint ROM for children in the United States of America (USA) provided by [7] was used for comparison with the current study. Left knee passive ROM data for children aged 2–12 years corresponding to the age group for the current study was used for comparison. This was done to ascertain the possible differences in knee ROM between children in the UAE and the USA, as ROM has been highlighted to be affected by cultural habits and daily life activities [3,5,18].

**Figure 1:**
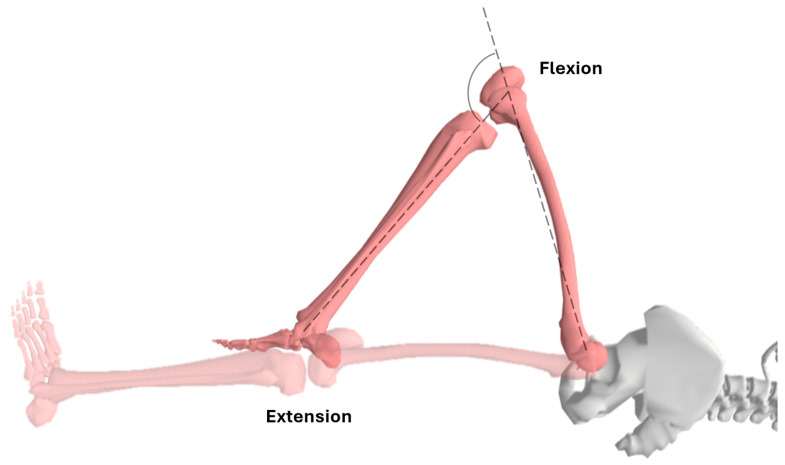
Lower extremity positions for knee range of motion assessment.

**Figure 2:**
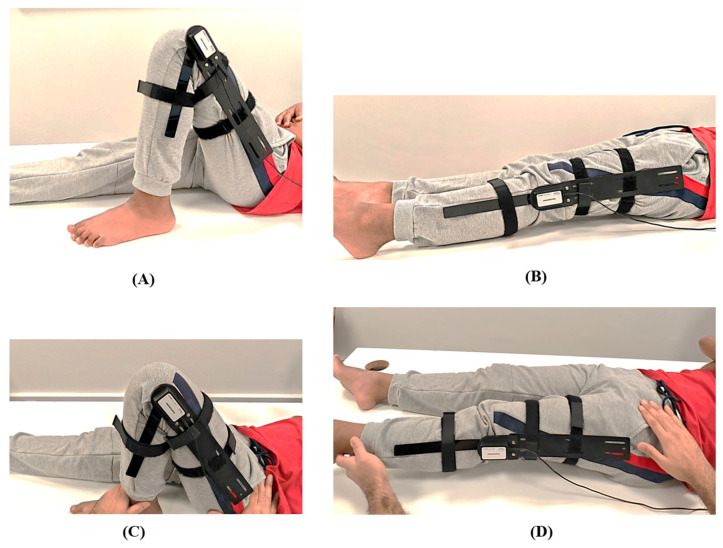
Knee range of motion assessment (**A**) active flexion, (**B**) active extension (**C**) passive flexion, (**D**) passive extension.

To measure knee flexion, the children were positioned supine with their knee and hip in extension. Their hip was ensured to be in 0° of adduction and abduction (Figure 1 and Figure 2). The hip was allowed to flex, but the femur was stabilized to prevent the adduction, abduction, and rotation of the hip. The end range was determined when additional hip flexion was caused as a result of resistance to further motion [46].

To measure knee extension, the children were also placed supine with their knee in full flexion and hip in 90° flexion (Figure 1 and Figure 2). Their hip was ensured to be in 0° of adduction and abduction, as was the case in knee flexion. The femur was stabilized to prevent hip adduction, abduction, and rotation. The end range was determined when the knee was passively extended by gravity [6].

The complete arc of motion of the knee joint was taken as the ROM (total extension + total flexion). A clinically significant difference was taken as any difference in ROM ≥ 10° as established by the American Medical Association and in previous studies [3,5,6,47,48].

### 2.4. Anthropometric Measurements

The anthropometric measurements for this study were obtained from the children based on the National Health and Nutrition Examination Survey (NHANES) guidelines for child anthropometric surveys [49]. The measurements taken from the children include body mass, height, waist circumference, buttock-popliteal length, thigh circumference, knee height, functional leg length, buttock-knee length, and thigh depth.

### 2.5. Data Analysis

Descriptive statistical analysis was conducted to characterize the study sample. The relationship between categorical variables was analyzed using either Pearson’s Chi-squared test or Fisher’s exact test as appropriate. Similarly, the association between categorical and numeric variables was examined using the Mann-Whitney U test, while the relationship between numeric variables was analyzed using Spearman’s correlation analysis. The significance level was set at 5%. All the analyses were performed using IBM SPSS software version 28. Reference values were established for each gender and age group.

## 3. Results

The demographic characteristics of the children are shown in Table 1. Most of the children were girls (52.5%) and from South Asia (53.2%). There was a significant difference in ethnicity between both genders (*p* < 0.001). The average age of the children was 8.1 years, with boys skewing significantly older than girls (*p* = 0.021). The average body mass index (BMI) for the children was 16.1, with no significant difference between boys and girls (*p* = 0.255).

For both girls and boys, knee flexion and ROM were greater in the children age group, while knee extension was higher in the adolescent age group for boys and greater in the children age group for girls (Table 2). The highest difference was observed in passive knee flexion, with about a 4° difference in the children age group compared to the adolescent age group. All other differences between the children and adolescent age groups were less than 4°. Small differences were observed in ROM between boys and girls (0.1–2.2°).

The comparison between passive and active knee ROM revealed passive flexion to be significantly higher than active flexion (*p* < 0.001), passive extension was significantly higher than active extension (*p* < 0.001), and PROM was significantly higher than AROM (*p* < 0.001) (Table 3). Passive (*p* < 0.001) and active (*p* = 0.005) knee flexion, PROM (*p* < 0.001), and AROM (*p* = 0.046), as well as active extension (*p* = 0.017) ROM were observed to be significantly higher among Arab children compared to South Asian children. No significant difference was observed in passive knee extension ROM (*p* = 0.401) between Arab and South Asian children, nor was any significant difference in knee ROM was observed between children and adolescents. Similarly, there was no significant difference in knee ROM between boys and girls (Table 4).

The comparison between children in the UAE and the USA in terms of demographic and anthropometric factors as well as passive knee ROM, revealed significant differences in age (*p* < 0.001), height (*p* < 0.001), and body mass (*p* < 0.001). Children in the USA have significantly higher passive knee flexion (*p* < 0.001) and PROM (*p* < 0.001), while children in the UAE have significantly higher passive knee extension ROM compared to children in the USA (*p* < 0.001) (Table 5). No significant difference in BMI (0.442) or gender (0.092) was observed between children in the USA and UAE.

Spearman’s correlation analysis between knee ROM and anthropometry revealed significant but weak negative correlation between passive (*r* = −0.218, *p* < 0.01) and active (*r* = −0.167, *p* < 0.01) flexion, PROM (*r* = −0.171, *p* < 0.01) and AROM (*r* = −0.151, *p* < 0.01) with body mass, passive (*r* = −0.174, *p* < 0.01) and active (*r* = −0.136, *p* < 0.01) flexion, PROM (*r* = −0.145, *p* < 0.05) and AROM (*r* = −0.136, *p* < 0.05) with thigh circumference, passive (*r* = −0.200, *p* < 0.01) and active (*r* = −0.134, *p* < 0.05) flexion, PROM (*r* = −0.152, *p* < 0.01) and AROM (*r* = −0.114, *p* < 0.05) with knee height, passive (*r* = −0.279, *p* < 0.01) and active (*r* = −0.246, *p* < 0.01) flexion, PROM (*r* = −0.238, *p* < 0.01) and AROM (*r* = −0.215, *p* < 0.01) with thigh depth, passive (*r* = −0.251, *p* < 0.01) and active (*r* = −0.224, *p* < 0.01) flexion, PROM (*r* = −0.224, *p* < 0.01) and AROM (*r* = −0.222, *p* < 0.01) with BMI, passive (*r* = −0.286, *p* < 0.01) and active (*r* = −0.238, *p* < 0.01) flexion, and PROM (*r* = −0.216, *p* < 0.01) and AROM (*r* = −0.186, *p* < 0.01) with waist circumference. Similarly, significantly weak negative correlations were observed between passive flexion with height (*r* = −0.152, *p* < 0.01), passive flexion with age (*r* = 0.139, *p* < 0.05), passive flexion with buttock-knee length (*r* = 0.130, *p* < 0.05), and passive (*r* = −0.171, *p* < 0.01) and active (*r* = −0.118, *p* < 0.05) flexion, and PROM (*r* = −0.118, *p* < 0.05) with functional leg length. No significant correlations were observed between knee extension ROM and anthropometry (Table 6).

## 4. Discussion

**Figure 3:**
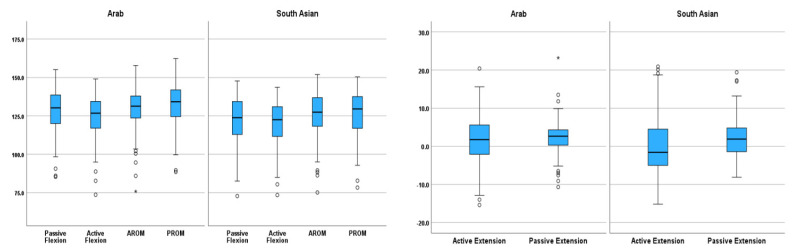
Knee ROM comparison between Arab and South Asian children. (The box represents the 25th to the 75th percentile IQR. The horizontal line within each box represents the median, the circles represent outliers while the asterisks represent extreme outliers).

**Figure 4:**
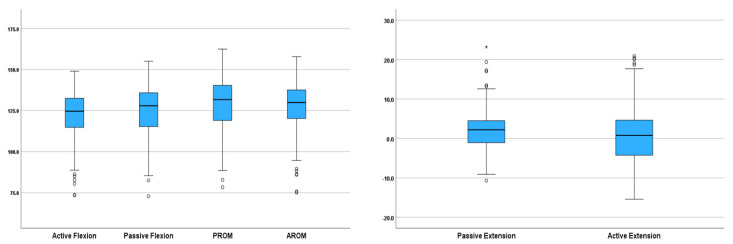
Differences in active and passive knee ROM in children. (The box represents the 25th to the 75th percentile IQR. The horizontal line within each box represents the median, the circles represent outliers while the asterisks represent extreme outliers).

This study established normative knee ROM for children and examined the effects of ethnicity, age, anthropometry, and gender on knee joint ROM in children. Statistically significant differences were observed in knee ROM between Arab and South Asian children (Figure 3) as well as active and passive ROM (Figure 4). However, none of the observed differences were clinically significant (<10°). Waist circumference, BMI, and thigh depth were observed as the most significant anthropometric parameters associated with knee joint ROM in children. These findings are consistent with the results of previous studies on normative knee ROM for children [1,2,4,7], as well as adults [3,5,6].

The reference values established in this study can serve as a guide for normal knee ROM values for a healthy child in the UAE. The normative values provided can also be useful for clinicians in the diagnosis of knee ROM deficiency for children in the UAE during clinical evaluations. The findings of this study can provide a basis for comparing normal knee ROM for children in the UAE. The results of the study can serve as a basis for assessing the response of children to rehabilitative interventions towards improving their knee ROM thereby aiding in the clinical decision-making process. Furthermore, the data presented can be used in the clinical evaluations of pediatric knee injuries and disorders in the UAE, thus serving as a tool for the screening and diagnosis of neuromuscular and musculoskeletal diseases. Moreover, the reference values established in this study can be used for measuring the efficacy of functional training programs aimed at restoring normal knee ROM for children in the UAE. All these clinical applications of ROM have been highlighted in previous studies [1,2,3,4,5,6,7].

Similar to the findings of our study, the studies by [1,4,8,9] also did not find any significant difference in knee joint ROM between boys and girls. To the best of our knowledge, this is the first study to examine the effects of ethnicity on joint ROM in children. Our findings showed significant differences in knee joint ROM between the two ethnicities studied. This highlights the need for normative reference values of extremity joint ROM for different populations. It should be noted that the South Asian category in our study included children from almost all South Asian countries, which together form about 25% of the world’s population. Additionally, the Arab category included children from several Arab countries, which together comprise about 5% of the world population [50]. In general, children from over twenty countries of origin participated in the study. We grouped them into two categories for ease of analysis and due to the fact that they can fit into a broader group. We excluded the “Other” category from the ethnic comparison due to the small sample size (8) and also due to the fact that they do not fit into a single ethnic composition. While we cannot claim that our results can be generalized to the larger global population, we strongly believe that our sample contained an adequate representation of the ethnicities that comprise the UAE population.

Knee joint ROM was observed to decrease significantly with increasing body mass, height, waist circumference, and BMI, which is consistent with the findings of [7] and [4], as the increase in BMI results in a decrease in joint mobility. The study by [4] also observed waist circumference to be the most significant predictor of joint ROM, as was the case in the current study. Tendon pathology has been identified to be associated with waist circumference due to systemic or mechanical effects, thereby affecting joint ROM [51,52]. We had intended to perform regression analyses to identify the anthropometric characteristics that relate to knee ROM and their relative contributions, but due to the very weak correlations between the anthropometric parameters and knee ROM in our study, we did not proceed with that idea. Previous studies that had employed regressions to identify the contribution of certain parameters to extremity joint ROM had usually used the threshold of correlation coefficient *r* ≥ 0.3 for the parameter to be included in the regression model [4]. In our own case, none of the correlations between the anthropometric parameters and knee ROM reached that threshold. Therefore, performing the regression analyses will not be justified, as the underlying assumptions for linear regression had not been fulfilled; there was no strong relationship between the anthropometric parameters and knee ROM in our study.

**Figure 5:**
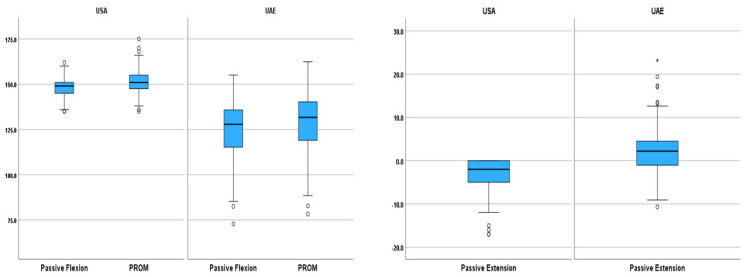
Passive knee ROM comparison between children in the USA and UAE. (The box represents the 25th to the 75th percentile IQR. The horizontal line within each box represents the median, the circles represent outliers while the asterisks represent extreme outliers).

The comparison between the results of this study and the publicly available dataset of extremity joints ROM provided by [7] revealed significant differences in age, body mass, height, as well as passive and active knee ROM between children in the USA and UAE. No significant differences were observed based on gender and BMI between the two study groups. The difference in passive knee flexion and PROM between the two study populations was clinically significant (>10°) (Figure 5).

The comparison between the results of this study and previous studies is presented in Table 7. Direct comparison with these studies is difficult due to the differences in sample size, gender, ethnicity, and age groupings. Additionally, we noticed differences between the methodology of the current study and the methodologies adopted in the previous studies, such as inclusion/exclusion criteria, sampling strategies, data analyses approaches, measurement protocols, and measurement instruments. Nevertheless, the values reported for knee joint ROM in all these studies are significantly higher (>10°) than the results of the current study. This indicates the need to use population-based reference values as differences in extremity joint ROM exist due to cultural habits and daily life activities [3,5,18].

Most of the studies on knee ROM measurements for children in the literature focused on children with some form of joint impairment [8,9,11,12,13,14,15,53,54,55,56,57,58]. The values obtained for knee flexion ROM in these studies ranged from 163° for healthy controls [9] to a preoperative value of 5° for children with severe knee extension stiffness [58]. On the other hand, knee extension ROM values ranged from −13° for children with hypermobility syndrome [8] to 10° for children with stiffness after knee surgery [56].

One interesting finding of this study is the fact that knee joint ROM in UAE children is significantly lower than even children with joint disorders in other countries. Children with juvenile idiopathic arthritis (139.1°) [9], hypermobility syndrome (155°) [8], and obesity (136.4°) [57] have significantly higher knee joint ROM compared to healthy children in the current study (127.9°). On the other hand, children with hemiplegic cerebral palsy (124.5°) [12] and knee stiffness (125°) [56] have comparable knee joint ROM with healthy children in the current study. Similarly, children with spastic diplegia (1.7°) [11] have comparable knee extension ROM with healthy children in the current study (2.2°).

It has been established in the literature that the method of ROM measurement affects the results [19,59]. For instance, warm up sessions before the measurement process had been found to increase ROM values by up to 6° [60]. Additionally, limb dominance is another factor that significantly affects ROM values [61]. Other factors, including the test position, test instrument, muscle mass, joint structure, muscle length, etc., have also been proven to affect ROM values [19,59,62,63]. For the purpose of consistency with our other ongoing studies (3D motion analysis and universal goniometry), only the left legs of the children were assessed using an electro-goniometer. We did not test for limb dominance, and it might be that the left limbs of the children in our study are not their dominant limb, thereby affecting the ROM values. Additionally, the instrument used in the current study differs with that of the other studies, which can also be a reason for the differences in results compared to previous studies for both typically developing children and children with disabilities. Furthermore, no warmup sessions were performed by the children in our study prior to the measurement process, as we believe that it may overestimate the actual joint ROM. Other factors like muscle mass, joint structure, and muscle length were not considered, as they were beyond the scope of our study.

Children in the UAE have extremely low levels of physical activity [64,65,66] and physical activity levels have been established to affect joint ROM [57,67]. This develops due to sedentariness and often leads to obesity, thereby resulting in decreased ROM [68,69,70]. This might also be the reason for the significantly lower knee joint ROM among children in the UAE compared to healthy children as well as children with joint impairments in other countries. However, information regarding the level and type of physical activity affecting joint mobility was not captured during the course of this study. This is one of the limitations of the current study and future studies should consider capturing this information in the data collection process. Studying the effect of physical activity levels on joint ROM is important not only to identify potential differences between groups, but also to explore variations between individuals. Such an investigation would require objective monitoring tools such as sensor-based activity monitors and would therefore constitute a separate study. If future studies identify significant variations in activity levels among children in the UAE, these findings should be considered, as they may add valuable context to the interpretation of extremity joint ROM outcomes.

The results of this study can be used in developing computational models of the pediatric knee for analyzing knee injuries due to vehicle crashes similar to [71], or knee joint injury mechanisms similar to [72], or knee joint impairments similar to [73].

## 5. Conclusions

In conclusion, this study presents the ROM of the pediatric knee for children in the United Arab Emirates (Arab and South Asian ethnicity) aged 3 to 12 years. The paper analyzed the effects of ethnicity, gender, anthropometry, and age on knee joint ROM in children. Significant differences in knee joint ROM were observed based on ethnicity but not age or gender. Our findings highlight the need for population-based reference values for extremity joint ROM in children. This will provide a better understanding of the ROM of the pediatric knee, thereby enhancing the assessment of knee injuries sustained by children and their associated risk factors.

## Figures and Tables

**Table 1:** Demographic characteristics of children in the study.

Characteristic	Gender				*p*-Value
	Girls		Boys		
	*N*	%	*N*	%	
Age Group					0.133
3–9	112	55.7%	89	44.3%	
10–12	43	45.7%	51	54.3%	
Ethnicity					<0.001
Arab	55	42.3%	75	57.7%	
South Asian	98	62.4%	59	37.6%	
Other	2	25.0%	6	75.0%	
Age (years), median (IQR)	8.0	(6.0, 10.0)	8.0	(7.0, 11.0)	0.021
BMI (kg/m^2^), median (IQR)	16.0	(14.3, 18.5)	16.2	(14.8, 19.0)	0.255

**Table 2:** Normative knee ROM for children in the UAE.

Knee ROM ^#^	Age Group	
	Children *	Adolescents *
Boys		
Passive Flexion	128.5 (114.3, 136.9)	125.7 (114.6, 135.4)
Active Flexion	124.9 (113.4, 132.9)	123.3 (114.8, 131.7)
Passive Extension	2.3 (−0.5, 3.9)	2.2 (−1.1, 5.4)
Active Extension	1.3 (−3.8, 5.2)	1.0 (−4.3, 6.8)
PROM	131.3 (116.5, 141.1)	129.9 (118.1, 139.3)
AROM	129.4 (118.4, 138.0)	128.2 (119.3, 137.1)
Girls		
Passive Flexion	129.6 (115.5, 136.8)	125.8 (116.9, 134.5)
Active Flexion	125.1 (115.4, 133.2)	125.1 (117.2, 131.7)
Passive Extension	1.9 (−2.0, 4.5)	2.8 (−1.1, 6.5)
Active Extension	0.5 (−4.7, 4.3)	1.7 (−5.0, 4.8)
PROM	133.5 (119.6, 141.7)	130.8 (123.0, 138.7)
AROM	131.0 (120.3, 137.4)	129.8 (123.2, 137.4)

* Median (IQR), ^#^ Degrees (°).

**Table 3:** Comparison between active and passive knee ROM in children.

	Active Extension Versus Passive Extension			Active Flexion Versus Passive Flexion			AROM Versus PROM		
	Active Extension *	Passive Extension *	*p*-Value	Active Flexion *	Passive Flexion *	*p*-Value	AROM *	PROM *	*p*-Value
Median (IQR)	0.8 (−4.3, 4.7)	2.2 (−1.1, 4.5)	<0.001	124.6 (114.8, 132.5)	127.9 (115.2, 135.9)	<0.001	129.9 (120.1, 137.7)	131.7 (118.9, 140.3)	<0.001

* Degrees (°).

**Table 4:** Knee ROM comparison based on age, gender, and ethnicity among children.

ROM *	Age Group		*p*-Value	Gender		*p*-Value	Ethnicity		*p*-Value
	Children	Adolescents		Girls	Boys		Arab	South Asian	
PF	128.7 (115.1, 136.8)	125.8 (115.4, 134.7)	0.282	128.5 (116.6, 136.0)	127.9 (114.5, 135.7)	0.546	130.2 (120.0, 138.6)	123.8 (112.9, 134.3)	<0.001
PE	2.1 (−1.0, 4.1)	2.8 (−1.1, 6.0)	0.152	2.4 (−1.4, 5.4)	2.2 (−0.4, 4.2)	0.978	2.7 (0.3, 4.3)	1.9 (−1.5, 4.9)	0.401
AF	124.9 (114.3, 133.1)	123.7 (115.6, 131.7)	0.747	125.1 (115.6, 132.5)	124.2 (113.5, 132.5)	0.594	126.8 (117.0, 134.5)	122.5 (111.6, 131.2)	0.005
AE	0.7 (−4.2, 4.6)	1.2 (−4.7, 5.2)	0.585	0.7 (−4.7, 4.4)	1.3 (−3.9, 5.3)	0.394	1.8 (−2.1, 5.7)	−1.6 (−5.1, 4.5)	0.017
PROM	132.2 (118.6, 141.2)	130.8 (119.9, 139.3)	0.528	132.8 (120.9, 140.3)	131.2 (117.2, 140.2)	0.194	134.2 (124.3, 142.0)	129.5 (116.9, 138.0)	<0.001
AROM	130.2 (119.5, 137.8)	128.6 (121.5, 137.4)	0.878	130.5 (120.9, 137.4)	129.3 (118.8, 137.9)	0.344	131.3 (123.3, 137.9)	127.4 (118.1, 137.1)	0.046

* Median (IQR), Degrees (°); PF: Passive Flexion; PE: Passive Extension; AF: Active Flexion; AE: Active Extension.

**Table 5:** Passive knee ROM comparison between children in the USA and UAE.

Parameter	Nationality		*p*-Value
	USA	UAE	
Age (Years) *	7.0 (4.0, 9.0)	8.0 (6.0, 10.0)	<0.001
Gender			0.092
Boys	78	140	
Girls	61	155	
Body Mass (kg) *	23.2 (17.3, 32.5)	27.7 (20.9, 36.7)	<0.001
Height (cm) *	122.0 (104.0, 137.0)	130.0 (119.1, 142.4)	<0.001
BMI (kg/m^2^) *	16.5 (15.2, 17.7)	16.1 (14.6, 18.7)	0.442
Passive Flexion *^,#^	149.0 (145.0, 151.0)	127.9 (115.2, 135.9)	<0.001
Passive Extension *^,#^	−2 (−5.0, 0.0)	2.2 (−1.1, 4.5)	<0.001
PROM *^,#^	151.0 (147.0, 155.0)	131.7 (118.9, 140.3)	<0.001

* Median (IQR), ^#^ Degrees (°).

**Table 6:** Correlation between knee ROM and lower extremity anthropometry.

Parameters	Passive Flexion	Passive Extension	PROM	Active Flexion	Active Extension	AROM
Age (years)	−0.139 *	0.109	−0.093	−0.065	0.057	−0.047
Body Mass (kg)	−0.218 **	0.080	−0.171 **	−0.167 **	0.080	−0.151 **
Height (cm)	−0.152 **	0.107	−0.104	−0.099	0.099	−0.077
BMI (kg/m^2^)	−0.251 **	0.026	−0.224 **	−0.224 **	0.042	−0.222 **
Waist Circumference (cm)	−0.286 **	0.065	−0.216 **	−0.238 **	0.079	−0.186 **
Buttock-Popliteal Length (cm)	−0.096	0.080	−0.079	−0.071	0.033	−0.066
Thigh Circumference (cm)	−0.174 **	−0.003	−0.145 *	−0.136 **	0.019	−0.136 *
Knee Height (cm)	−0.200 **	0.075	−0.152 **	−0.134 *	0.063	−0.114 *
Functional Leg Length (cm)	−0.171 **	0.113	−0.118 *	−0.118 *	0.081	−0.096
Buttock-Knee Length (cm)	−0.130 *	0.088	−0.083	−0.070	0.048	−0.044
Thigh Depth (cm)	−0.279 **	0.021	−0.238 **	−0.246 **	0.045	−0.215 **

** *p* < 0.01; * *p* < 0.05.

**Table 7:** Comparison of normative knee ROM with previous studies.

Study	Sample Size	Age Group	Passive Flexion ^#^	Passive Extension ^#^	Active Flexion ^#^	Active Extension ^#^
Current study	295	3–12	125.5(14.0) *	2.3(4.7) *	122.6(13.4) *	0.9(6.5) *
Current study	123	3–7	128.1(12.7) ^c^	1.7(4.2) ^c^	123.8(13.6) ^c^	0.4(5.9) ^c^
Current study	172	8–12	123.7(14.7) ^d^	2.7(5.0) ^d^	121.7(13.4) ^d^	1.3(6.9) ^d^
[1]	34	6–16	156.6(4.1) *	4.3(4.4) *	-	-
[1]	16	6–16	158.0(3.2) ^a^	4.4(4.4) ^a^	-	-
[1]	18	6–16	155.4(4.6) ^b^	4.2(4.5) ^b^	-	-
[7]	39	2–8	152.6(4.5) ^a^	5.4(4.9) ^a^	-	-
[7]	55	2–8	147.8(4.7) ^b^	1.6(2.5) ^b^	-	-
[7]	56	9–19	142.3(5.7) ^a^	2.4(3.5) ^a^	-	-
[7]	48	9–19	142.2(6.3) ^b^	1.8(3.2) ^b^	-	-
[2]	53	4–16	-	4.0(5.0)*	-	-
[2]	20	4–7	-	5.0(4.0) ^c^	-	-
[2]	17	8–11	-	4.0(6.0) ^d^	-	-
[2]	16	12–16	-	1.0(4.0) ^e^	-	-
[4]	70	3–9	-	-	145.0(5.5) ^b^	4.0(3.3) ^b^
[4]	70	3–9	-	-	144.0(5.7) ^a^	4.0(3.9) ^a^
[4]	80	10–19	-	-	140.0(6.7) ^b^	2.0(2.6) ^b^
[4]	80	10–19	-	-	142.0(6.6) ^a^	2.0(2.6) ^a^
[10]	22	6–17	-	1.4(6.4) ^f^	-	-
[10]	22	6–17	-	−3.6(3.6) ^g^	-	-

* Entire sample; ^a^ Girls; ^b^ Boys; ^c^ Children; ^d^ Adolescents; ^e^ Teenagers; ^f^ Spastic Diplegia; ^g^ Controls, ^#^ Degrees (°).

## Data Availability

Data will be made available upon request through the corresponding author.
